# Novel Type III Polyketide Synthases Biosynthesize Methylated Polyketides in *Mycobacterium marinum*

**DOI:** 10.1038/s41598-018-24980-1

**Published:** 2018-04-25

**Authors:** Amreesh Parvez, Samir Giri, Gorkha Raj Giri, Monika Kumari, Renu Bisht, Priti Saxena

**Affiliations:** 1 Chemical Biology Group, Faculty of Life Sciences and Biotechnology, South Asian University, New Delhi, 110021 India; 2 Present Address: Department of Ecology, School of Biology, University of Osnabrück, Osnabrück, 49076 Germany; 3 Present Address: Department of Biochemistry, University College of Medical Sciences, Delhi, 110095 India

## Abstract

Mycobacterial pathogenesis is hallmarked by lipidic polyketides that decorate the cell envelope and mediate infection. However, factors mediating persistence remain largely unknown. Dynamic cell wall remodeling could facilitate the different pathogenic phases. Recent studies have implicated type III polyketide synthases (PKSs) in cell wall alterations in several bacteria. Comparative genome analysis revealed several type III *pks* gene clusters in mycobacteria. In this study, we report the functional characterization of two novel type III PKSs, MMAR_2470 and MMAR_2474, in *Mycobacterium marinum*. These type III *pks*s belong to a unique *pks* genomic cluster conserved exclusively in pathogenic mycobacteria. Cell-free reconstitution assays and high-resolution mass spectrometric analyses revealed methylated polyketide products in independent reactions of both proteins. MMAR_2474 protein exceptionally biosynthesized methylated alkyl-resorcinol and methylated acyl-phloroglucinol products from the same catalytic core. Structure-based homology modeling, product docking, and mutational studies identified residues that could facilitate the distinctive catalysis of these proteins. Functional investigations in heterologous mycobacterial strain implicated MMAR_2474 protein to be vital for mycobacterial survival in stationary biofilms. Our investigations provide new insights into type III PKSs conserved in pathogenic mycobacterial species.

## Introduction

Mycobacteria genus comprises of some of the deadliest pathogenic species^1^. While *Mycobacterium tuberculosis* (Mtb) and *Mycobacterium leprae* have been banes of humankind, members of related species are known to infect other organisms, including cattle, birds, fish and other marine hosts^2^. *Mycobacterium marinum* (Mmar), the water-dwelling mycobacteria, causes systemic granulomas and lesions in fish and frog and is the causative agent of aquarium granulomas in humans. Mmar shares 85% genome-wide amino acid sequence identity with Mtb and is a model organism for dissecting mycobacterial pathogenesis^3^. Last two decades have seen tremendous progress in our understanding of mycobacterial virulence^4–7^. Mega-synthetic polyketide synthases (PKSs), in conjunction with fatty acid synthases, generate a repertoire of polyketide lipids that surface Mtb cell envelope and aid virulent phases of tuberculosis^6^. Efforts, however, are on to dissect molecular mechanisms that mediate mycobacterial persistence^4,8–10^.

Comparative genome analyses has revealed conservation of several biosynthetic *pks* genes across various mycobacterial species^11,12^. Many of these *pks*s are clustered in biosynthetic operons and are uniquely conserved in genomes of pathogenic mycobacteria. Mmar genome harbors 34 ORFs homologous to *pks* genes, with four being type III *pks*s^3^. Type III polyketide synthases have shown immense potential to biosynthesize architecturally diverse and bio-functionally distinctive metabolites^13–19^. These small, homodimeric proteins utilize a single catalytic cysteine residue in each monomer and iteratively condense activated coenzyme-A (CoA) analogs of mono-carboxylic aliphatic/aromatic starter substrates with repeating di-carboxylic malonyl-/methylmalonyl-CoA extender units^20–22^. It is interesting to note that these proteins can cyclize common reaction intermediates through at least three chemically distinct mechanisms, leading to an array of products with varied chemical scaffolds^23,24^. Substrate promiscuity, variable iterations, and multiple cyclization potential confer these proteins with the ability to generate a palette of diverse products utilizing limited substrate pool^17,22,23,25–31^. Variability is further generated by post-synthesis modifications of the polyketide products by other modifying enzymes that impart biological activity to the nascent polyketide cores^32^. Notably, genes for polyketide modifying enzymes are known to be clustered with type III *pks* genes in microbial genomes^15,33,34^. Several of these type III *pks* clusters have been recently implicated in generating functionally important metabolites. *srs* cluster in *Streptomyces griseus* produces methylated polyketides that confer antibiotic resistance to the species^34^. Phenolic lipids in *Azotobacter vinelandii* are biosynthesized by a cluster comprising of type I *pks*s and two type III *pks*s^35^. These molecules replace membrane phospholipids during encystment of dormant cells and are important components of cyst exine^35^. Recently, methylated polyketide quinones from *Mycobacterium smegmatis* (Msmeg) have been shown to facilitate anaerobic respiration of Msmeg cells in stationary biofilms^36^. These molecules are produced by a genomic cluster of Msmeg homologous to the *srs* operon. While an orthologous genomic cluster is not present in Mtb, polyketide quinones were isolated from Mtb biofilms and a homologous type III PKS was implicated in their biosynthesis. Growth in multicellular aggregates, like biofilms, provides a secure niche for developing mycobacterial persistence and advantages of multi-drug resistance^37,38^.

Here, we report the functional characterization of two novel type III PKSs from Mmar. These type III *pks*s belong to *Mycobacterium* specific 2x type III *pks* genomic cluster conserved exclusively in pathogenic species. MMAR_2470 and MMAR_2474 proteins biosynthesized methylated polyketide metabolites from acyl-CoA starter units selectively utilizing two different extender substrates. Distinctively, MMAR_2474 protein catalyzed the biosynthesis of methylated alkyl-resorcinols and methylated acyl-phloroglucinols from the same protein core. Homology-based structural modeling, molecular docking and site directed mutagenesis identified catalytically crucial active site residue positions and revealed clues to the structure-function relationship in Mmar type III PKSs. High-resolution metabolomic investigations of heterologous complemented type III *pks* knockout mycobacterial strain revealed the physiological importance of methylated polyketides and implicated the biosynthetic proteins in aiding mycobacterial survival in biofilms.

## Results

### Genomic conservation of 2x type III *pks* cluster in pathogenic mycobacteria

**Figure 1:**
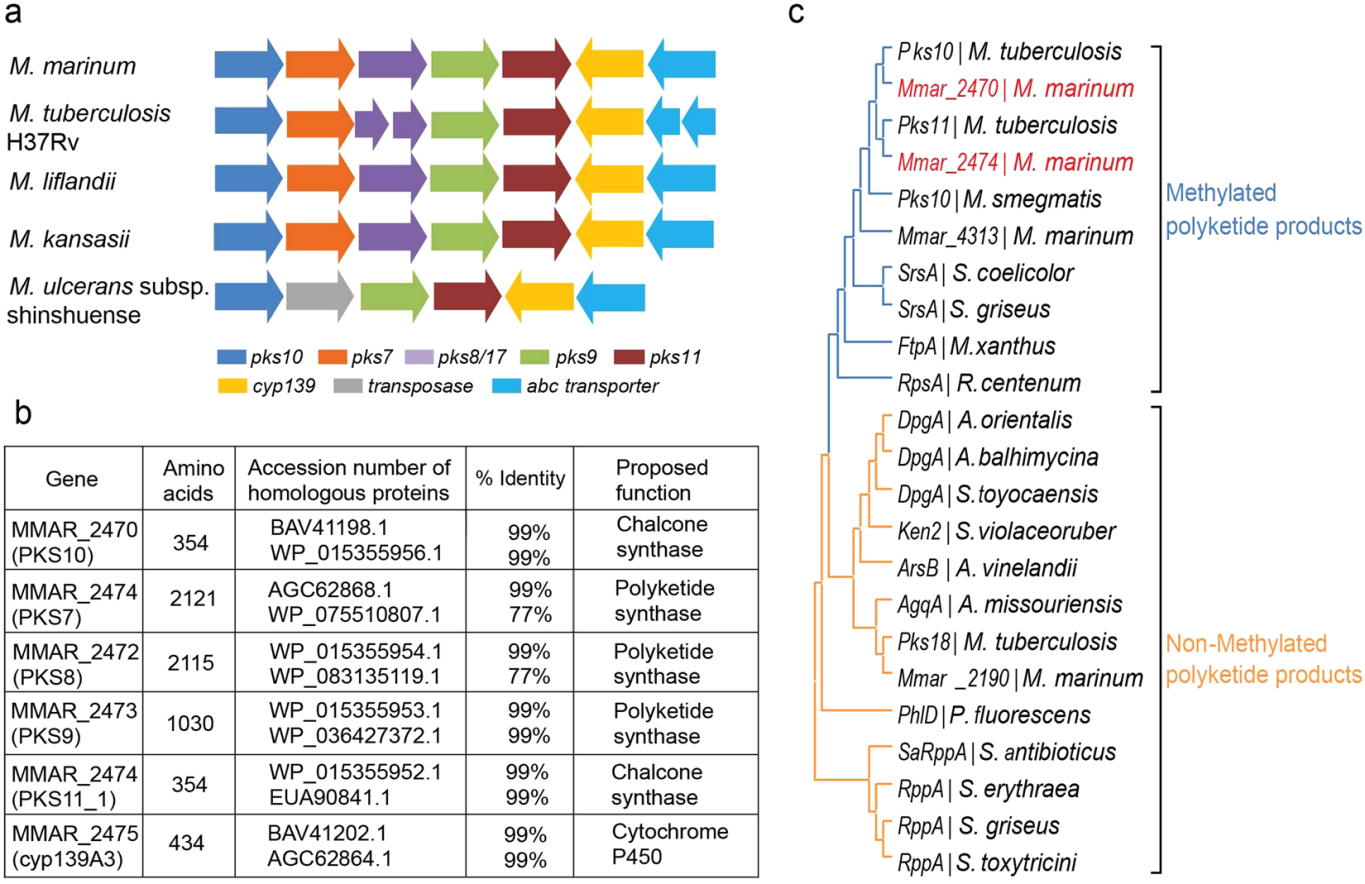
Conservation of 2x type III *pks* genomic cluster and phylogenetic analysis of *M. marinum* (Mmar) type III PKSs. (**a**) Conservation of the 2x type III *pks* genomic cluster in pathogenic mycobacterial species. (**b**) Sequence analysis of Mmar 2x type III *pks* genomic cluster. (**c**) Phylogenetic analyses of Mmar type III PKS proteins with functionally characterized microbial type III PKSs. Type III PKSs biosynthesizing non-methylated polyketides are represented in *orange* color, while type III PKSs biosynthesizing methylated polyketide products are represented in *blue* color. The Mmar type III PKSs discussed in this study are highlighted in *red* color. The source organism is mentioned along with the protein names.

Mycobacterial genome sequencing has revealed a plethora of genes homologous to polyketide synthases (*pks*s). Many of these *pks*s are clustered with other polyketide related genes in the genomes. Functional characterization of several *pks* clusters in Mtb in the last two decades have implicated several multi-domain, mega-synthetic type I PKSs in the biosynthesis of unique virulent mycobacterial polyketide lipids^6^. Lately, type III polyketides from diverse microorganisms have exhibited remarkable functional capabilities. Comparative genome analyses revealed novel type III *pks* clusters in several mycobacterial species. We investigated the occurrence of type III *pks* genes in Mmar. This organism shares substantial number of conserved genes with human pathogen Mtb^3,39^. Genome sequencing identified four type III *pks* genes in Mmar. Interestingly, three of these genes, *mmar_2190*, *mmar_2470* and *mmar_2474* were exclusively conserved in pathogenic mycobacteria whereas; the fourth homolog, *mmar_4313*, could be identified in several environmental mycobacteria. The *mmar_4313* genomic cluster of Mmar is orthologous to the polyketide quinone biosynthetic gene cluster recently characterized in Msmeg^36^. *mmar_2190* is an ortholog of alkyl pyrone biosynthesizing *pks18* gene of Mtb. The *mmar_2470* and *mmar_2474* genes constitute a unique genomic cluster in Mmar as shown in Fig. 1a. This gene cluster contains three multi-functional type I *pks* genes flanked on either side by the type III *pks*s. The cluster shows conservation of cytochrome P450 and ABC transporter genes downstream of *mmar_2474*. It is interesting to note that an orthologous 2x type III *pks* genomic organization, though identified to be non-essential in Mtb genome^40,41^, is exclusively conserved in almost all pathogenic mycobacteria with 77–99% amino acid sequence identity (Fig. 1b), suggesting the possibility of its involvement in mycobacterial pathogenesis.

Type III PKSs demonstrate promiscuity in substrate selection, leading to variability in chemical profiles of the biosynthesized polyketide products. A number of iterative condensations of growing polyketide chain and cyclization chemistry facilitated by the protein to generate biosynthesized products further augment product diversity. We attempted to predict the biosynthetic potential of MMAR_2470 and MMAR_2474 proteins by comparing these with experimentally characterized bacterial type III PKSs. Figure 1c shows a phylogenetic tree constructed using amino acid sequences of 23 bacterial type III PKS proteins exhibiting varied enzymatic catalysis. It is interesting to note that sequences cluster in three distinct groups, based on substrate preference and chemical architecture of the biosynthesized products. Proteins with the ability to condense several molecules of di-carboxylic malonyl-CoA units, without requirement of a mono-carboxylic starter substrate, are closely placed in phylogenetic tree, next to the group that represents sequences of proteins with a specific preference for malonyl-CoA as extender substrate. Proteins in both these groups catalyze formation of non-methylated polyketide scaffolds. It can be noted that of the four type III PKSs of Mmar, MMAR_2190 protein groups with malonyl-CoA accepting proteins, while the other three Mmar type III PKSs distinctly cluster with proteins that biosynthesize methylated polyketide products. These C-methylated polyketides are biosynthesized by incorporation of methylmalonyl-CoA along with malonyl-CoA extender units during polyketide chain elongation. Our phylogenetic analyses could reliably group Mmar proteins along with their orthologous sequences from other species and provides clues to the substrate preference and product profiles of the Mmar type III PKS proteins. Based on these analyses we predicted that MMAR_2470 and MMAR_2474 proteins would exhibit the potential to utilize both malonyl- and methylmalonyl-CoA extender units and biosynthesize methylated polyketide metabolites.

### Homology modeling and structure/function analyses of Mmar type III PKSs

**Figure 2:**
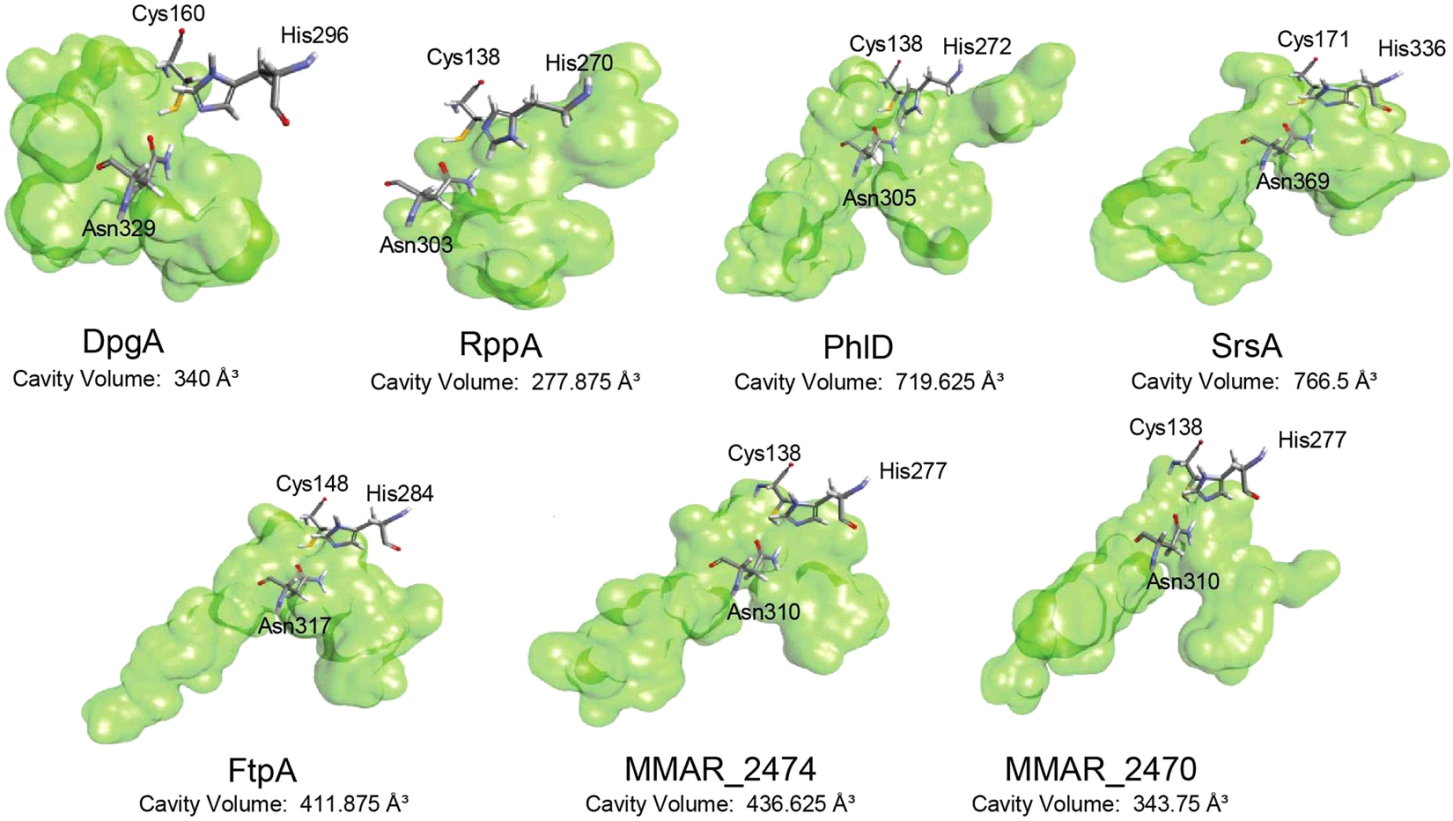
Homology modeling and comparison of ligand/product binding cavities of MMAR_2470 and MMAR_2474 proteins with experimentally characterized bacterial type III PKSs. Homology models of the type III PKSs were generated using Biovia Discovery Studio 4.5. The homology models along with x-ray crystallographic structure of RppA (PDB id 1U0M_B) were probed for differences in architecture and volumes of the product binding cavities in each protein. The *Cys*, *His* and *Asn* catalytic triad is shown along with the identified cavity. Cavity volumes (in Å^3^) are mentioned below the protein names.

We further investigated the possibility of biosynthesis of methylated metabolites by mycobacterial proteins using structure-based homology modeling. Three-dimensional homology models were generated for MMAR_2470 and MMAR_2474 proteins using the x-ray crystallographic structure of Mtb PKS11 protein (4JAQ_D)^42^ as template. Functionally characterized FtpA, DpgA, PhlD and SrsA type III PKSs from *M. xanthus*^43^, *S. lividans*^44^, *P. fluorescens*^45^ and *S. griesus*^18^, respectively, were modeled using closest structural homolog for each. Details of templates used for generating each model and the calculated model energies are provided in Supplementary Table S1. Computationally generated models along with the crystal structure of THNS protein (1U0M_B) from *S. erythraea*^20^ were probed for differences in volume capacity of the product binding cavities in core of the proteins. As can be observed from Fig. 2, the product binding cavities of MMAR_2470 and MMAR_2474 are strikingly similar to the cavity of FtpA protein. FtpA utilizes both malonyl- and methylmalonyl-CoA extender units and synthesizes methylated phenolic lipids with long-chain acyl-CoA starter molecules. Based on our *in silico* analyses, we speculated that MMAR_2470 and MMAR_2474 proteins would be able to utilize methylmalonyl-CoA as an extender unit along with malonyl-CoA and biosynthesize methylated polyketide products.

### Biochemical characterization of Mycobacterial type III PKSs

**Figure 3:**
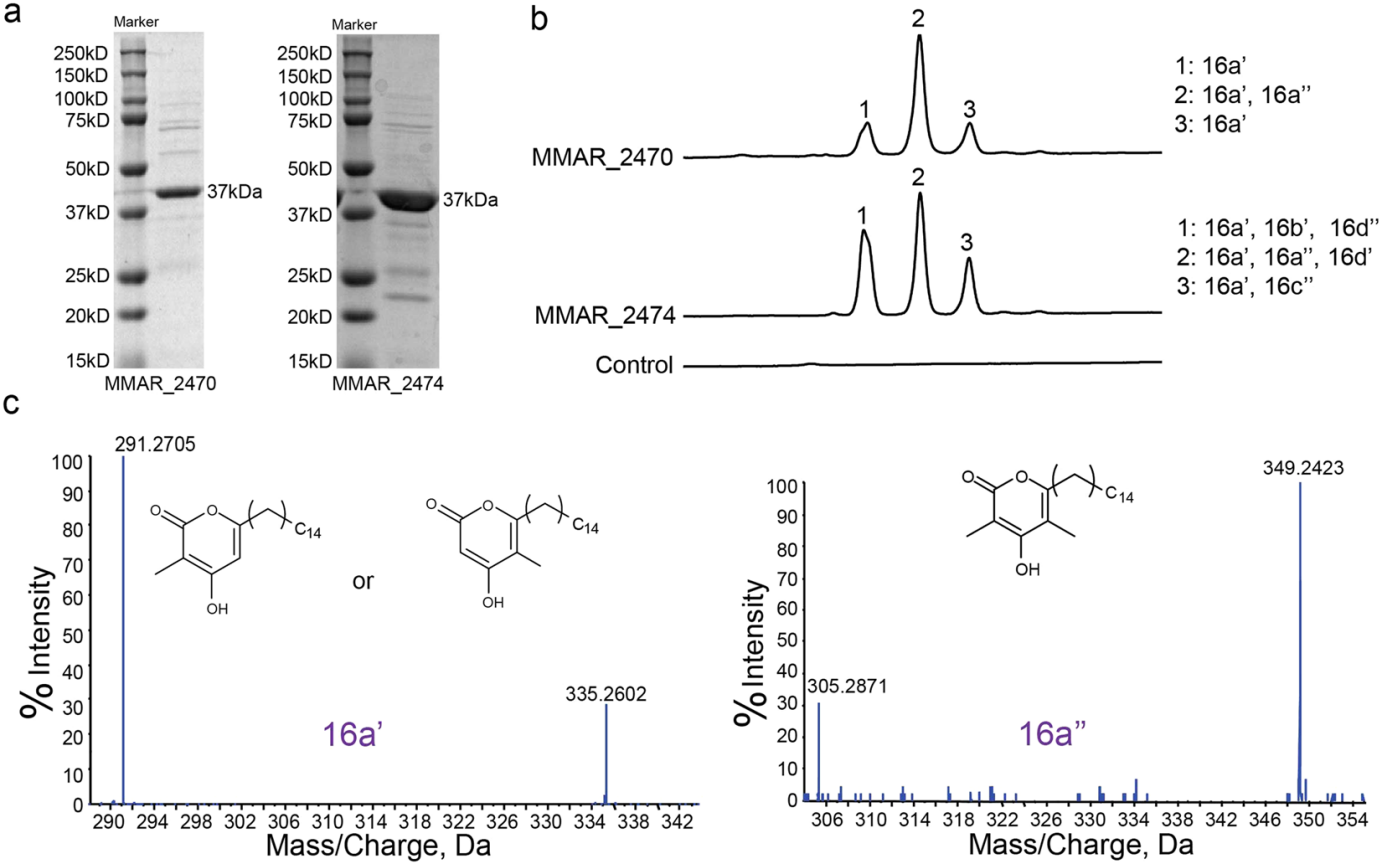
Biochemical analyses of MMAR_2470 and MMAR_2474 proteins and mass spectrometric characterization of reaction products. (**a**) SDS-PAGE profiles of purified MMAR_2470 and MMAR_2474 proteins. (**b**) UFLC chromatograms at 280 nm for reaction products of enzymatic assays carried out independently for both the Mmar type III PKS proteins. Reactions were primed with palmitoyl-CoA (C_16_-CoA) as starter substrate and both malonyl- and methymalonyl-CoA as extender substrates. Control chromatogram represents reaction carried out in the absence of Mmar proteins. (**c**) Tandem MS/MS analysis of reaction products of MMAR_2470 protein.

The two type III PKSs, MMAR_2470 and MMAR_2474 proteins in Mmar, share 74% sequence identity with each other. We cloned the *mmar_2470* and *mmar_2474* genes from genomic DNA of Mmar and over-expressed these proteins in *Escherichia coli* under the control of T7 expression system. The recombinant hexa-histidine-tagged proteins were purified by Ni^2+^-nitrilotriacetic acid affinity chromatography as single protein bands of ~37 kDa each, as determined by SDS-PAGE (Fig. 3a).

**Figure 4:**
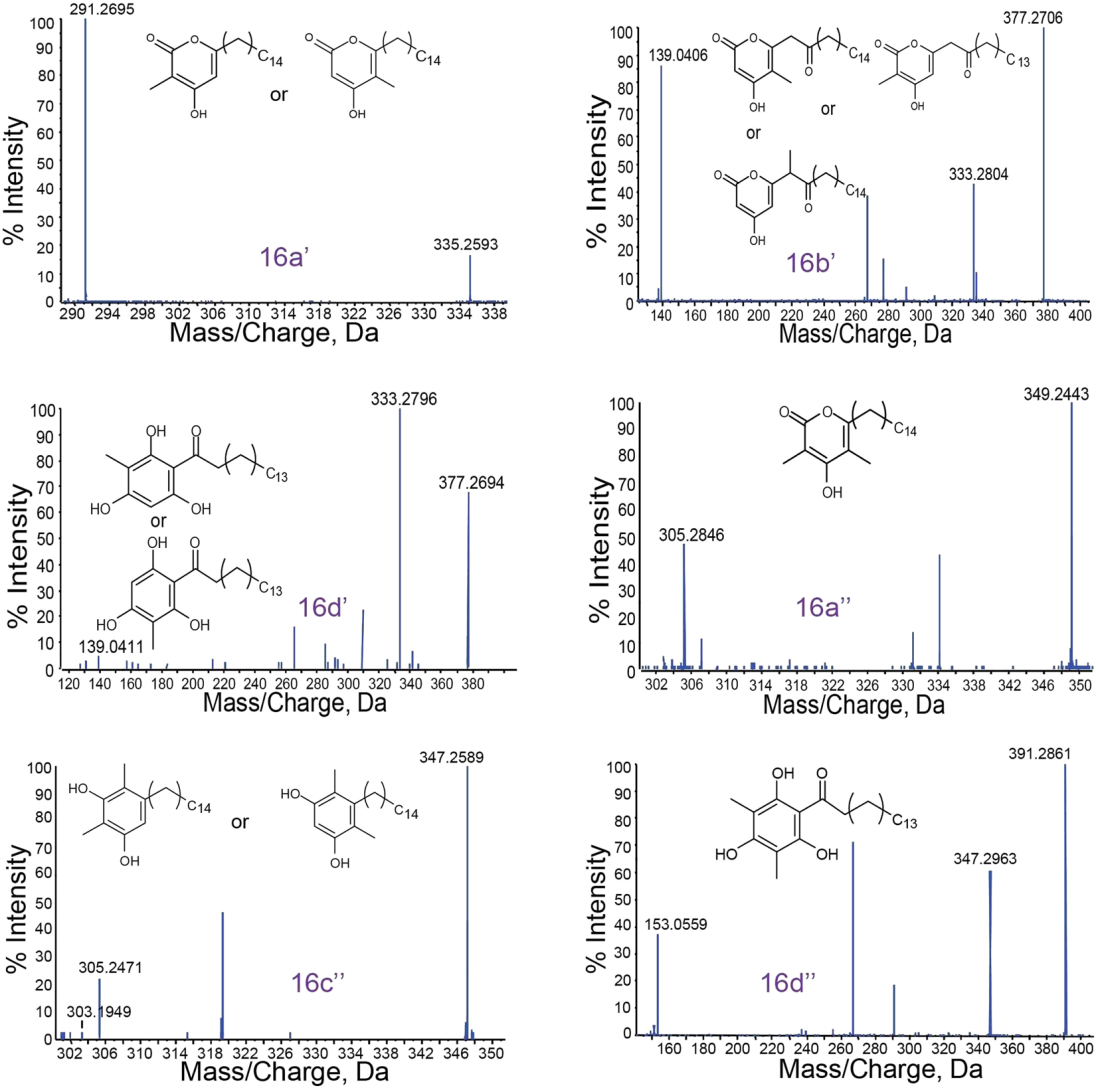
Triple-TOF HRMS identification of novel methylated tetraketide reaction products of MMAR_2474 protein. Tandem MS/MS spectra for each product of MMAR_2474 protein from assays carried out with palmitoyl-CoA (C_16_-CoA) as starter substrate and both malonyl- and methymalonyl-CoA as extenders.

Our *in silico* phylogenetic analyses of MMAR_2470 and MMAR_2474 proteins showed a preference of these mycobacterial proteins to group with FtpA, SrsA, and Msmeg PKS10 proteins. Notably, these latter iterative type III PKSs display potentials to selectively condense malonyl- and methylmalonyl-CoA units as extender substrates with variable acyl-CoA starter units to generate a series of methylated polyketide products. We investigated biochemical functions of the two mycobacterial proteins by carrying out cell-free enzymatic assays. Purified recombinant MMAR_2470 and MMAR_2474 proteins were incubated independently with malonyl-CoA and/or methylmalonyl-CoA as extender substrates in the presence of long-chain acyl-CoA starter units. Reaction products were extracted and separated using ultra-fast liquid chromatography (UFLC) and chemically characterized by high-resolution mass spectrometry (HRMS). Mass spectrometric analyses corroborated our computational studies and revealed the identity of methylated products for both mycobacterial proteins. Mycobacterial MMAR_2470 and MMAR_2474 proteins exhibited the ability to utilize malonyl- or methylmalonyl-CoA alone as extender units to mainly biosynthesize non-methylated and di-methylated triketide α-pyrone products utilizing each extender unit (Supplementary Figure S1, Supplementary Tables S2, S3). It was interesting to note that reactions with both malonyl- and methylmalonyl-CoA as extender units resulted in more than one reaction products. The UFLC chromatogram of enzymatic assays primed with palmitoyl-CoA (C_16_-CoA) and a combination of malonyl- and methylmalonyl-CoA can be visualized in Fig. 3b. The UFLC profiles for reactions with MMAR_2470 and MMAR_2474 proteins showed three product peaks **1**, **2** and **3** at 280 nm in each case. The product from each peak was subjected to HRMS analyses. In the MMAR_2470 reaction, a molecular ion peak of [M-H]^−^ at *m/z* 335.2602 could be observed in all three chromatographic peaks, while peak **2** in the chromatogram revealed an additional molecular ion of [M-H]^−^ at *m/z* 349.2423. A tandem MS/MS analyses confirmed identities of the two products as mono- and di-methylated palmitoyl-triketide α-pyrones, 16a’ and 16a”, respectively (Fig. 3c, Supplementary Table S2). Mass spectrometric identification of similar molecules has been previously reported for reaction products of PKS10 and RpsA proteins from Msmeg and *R. centenum*, respectively^36,46^. Both these products could be identified in the three chromatographic peaks observed for MMAR_2474 reactions. In addition to the ability of MMAR_2470 protein to extend starter units with two rounds of condensations to form triketide products, MMAR_2474 protein performed three rounds of condensations with the extender units to generate tetraketide products. Molecular ion peaks of [M-H]^−^ at *m/z* 377.2706 in peak 1 and [M-H]^−^ at *m/z* 347.2589 in peak 3 could be expected from a mono-methylated palmitoyl-tetraketide α-pyrone (16b’) and a di-methylated palmitoyl-resorcinol (16c”) molecule, respectively. The identity of the products was confirmed using tandem MS/MS analyses as shown in Fig. 4 and Supplementary Table S3. To our surprise, we observed two additional molecular ions of [M-H]^−^ at *m/z* 377.2694 and *m/z* 391.2861, respectively, in peaks 2 and 1 of the chromatogram. Tandem MS/MS analysis confirmed the identity of these molecular masses as mono- and di-methylated palmitoyl-phloroglucinols, 16d’ and 16d” (Fig. 4, Supplementary Table S3). Mass spectrometric characterization of non-methylated acyl-phloroglucinols has previously been reported for products of RppA protein from *S. griseus*^29^. The concomitant biosynthesis of methylated alkyl-resorcinol and methylated acyl-phloroglucinol from the same catalytic pocket is a unique observation in the microbial type III PKS protein family. Our biochemical analyses of MMAR_2470 and MMAR_2474 proteins from Mmar establish the biosynthetic functions of these mycobacterial proteins.

### Computational docking studies provide clues to unique bio-functionality of Mmar Type III PKSs

**Figure 5:**
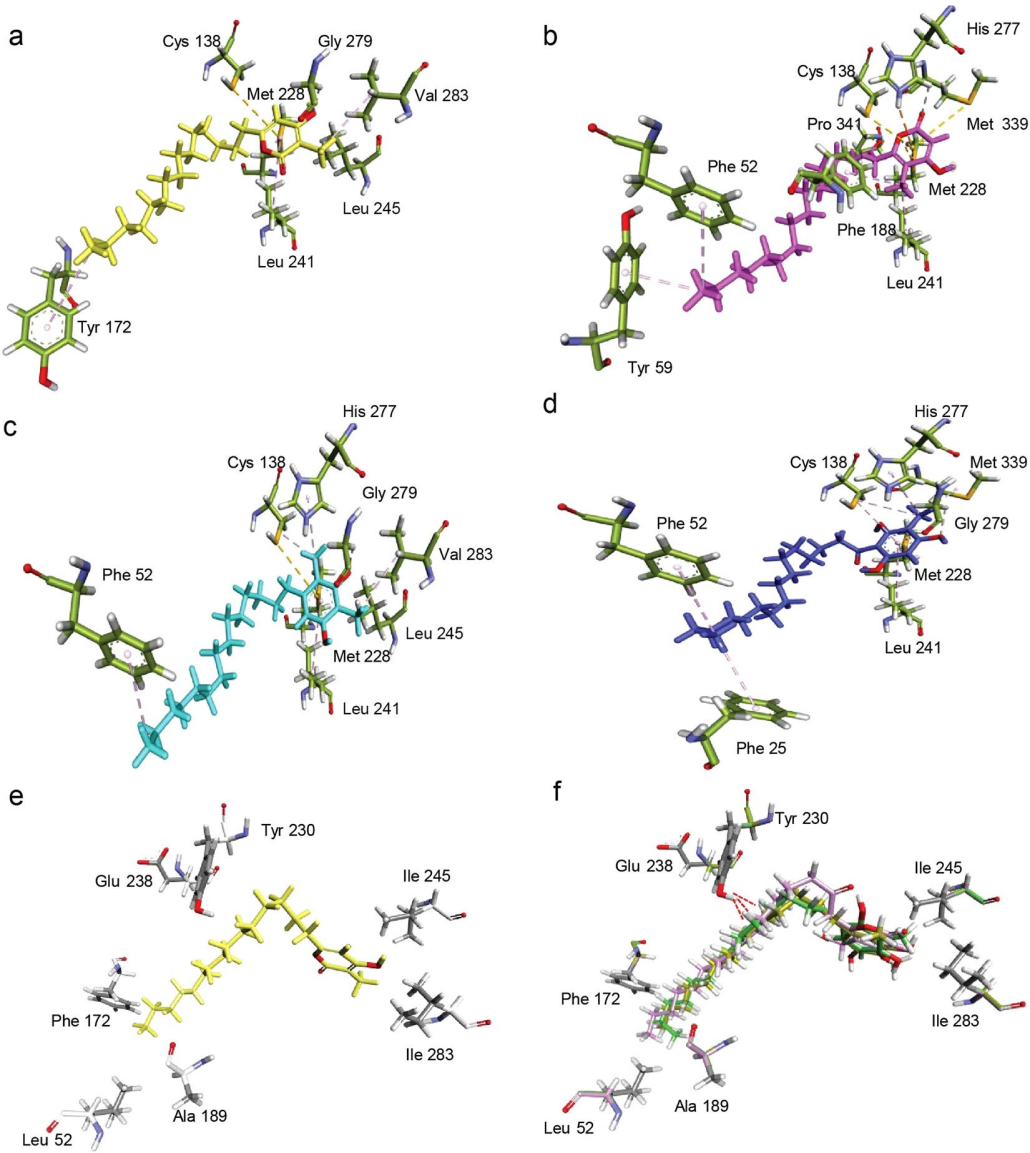
Protein-ligand/product docking studies to delineate structural basis for cyclization specificity in mycobacterial type III PKSs. The products of MMAR_2474 protein were docked in MMAR_2474 homology model. Docking was performed with (**a**) mono-methylated triketide α-pyrone (16a’) (**b**) mono-methylated tetraketide α-pyrone (16b’), (**c**) di-methylated alkyl-resorcinol (16c”) and (**d**) mono-methylated acyl-phloroglucinol (16d’). Interactions with amino acid side chains of MMAR_2474 protein are shown in each case. To identify the structural basis for lack of tetraketide products from MMAR_2470 protein, the cavity lining residues in the MMAR_2474 model were systematically replaced with corresponding amino acid residues in MMAR_2470 protein. (**e**) Shows a mono-methylated triketide α-pyrone (16a’) docked without clashes in the reduced cavity of the mutated MMAR_2474 model. (**f**) Replacement of *Trp* at position 230 in MMAR_2474 with corresponding *Tyr* in MMAR_2470 results in a clash with all three tetraketide products (16b’, 16c” and 16d’) docked in the cavity.

Biochemical characterization of Mmar type III PKSs revealed that while both proteins actively biosynthesized mono- and di-methylated products, MMAR_2474 protein exhibited remarkable ability to synthesize methylated alkyl-resorcinols and methylated acyl-phloroglucinols in the same reaction. We probed product formation by docking biochemically identified polyketide products of MMAR_2474 on to the three-dimensional homology model generated for this protein. Figure 5a–d, respectively show docked 16a’, 16b’, 16c” and 16d’ reaction products and interacting side chains of cavity lining residues with each of the products. As can be observed from the figure, docked products displayed a variation in the orientation of the alkyl-chain placed in substrate-binding tunnel, leading to differences in interacting residues of the MMAR_2474 protein. List of interacting residues with each docked substrate is given in Supplementary Table S4. The alkyl-chains showed interactions with hydrophobic *Tyr* and *Phe* residues of the substrate-binding tunnel. These interactions could stabilize the tetraketide intermediate in an orientation necessary for each type of cyclization to occur. We noticed that a specific *Phe*52 residue in the substrate-binding tunnel showed interaction with all three tetraketide products, 16b’, 16c” and 16d’ docked in MMAR_2474 protein. Interestingly, this *Phe*52 residue is uniquely present in the substrate-binding tunnel in MMAR_2474 protein. The corresponding position in functionally related microbial type III PKSs is occupied by a conserved *Leu* residue (Supplementary Figure S2). It is tempting to speculate that the interactions of growing polyketide intermediate with *Phe*52 residue in substrate-binding pocket in MMAR_2474 protein stabilize an orientation of the tetraketide intermediate that facilitates cyclization by three cyclization chemistries, leading to methylated alkyl-tetraketide α-pyrone, methylated alkyl-resorcinol and methylated acyl-phloroglucinol formation. During dynamic functioning of the protein, additional hydrophobic interactions with *Tyr*59 or *Phe*25 might aid the tetraketide intermediate in undergoing, respectively, lactonization or claisen condensation in the cyclization pocket. As can be observed from the multiple sequence alignment in Supplementary Figure S2, *Tyr* at position 59 in MMAR_2474 is conserved in the methylated tetraketide α-pyrone forming proteins. Our docking studies revealed differences in placement and interactions of cyclized ring system of all docked reaction products in the cyclization pocket. A close inter-link between the substrate-binding pocket and the cyclization cavity has been previously known in PKSIII*Nc* protein from *Neurospora crassa* to influence orientation of the growing polyketide intermediates and dictate cyclization chemistry of reaction products^47^. A close examination of active site residues revealed a *Trp* residue at position 230 in MMAR_2474 protein. *Trp* at the same position in ArsB protein from *Azotobacter* has been remarkably shown to be essential for biosynthesis of resorcinol products^35^. Conservation of *Trp* at this position has been reported for functionally characterized alkyl-resorcinol forming, bacterial FtpA, SrsA, PKS10 and PKS11 proteins from *M. xanthus*^43^, *S. griseus*^18^, Msmeg and Mtb^36^, respectively.

MMAR_2470 protein displayed a restricted product profile and biosynthesized methylated alkyl-triketide α-pyrones as the sole catalytic product, a property not yet reported for microbial type III PKSs. Gene inactivation studies recently implicated CPZ6, a type III PKS from *S. coelicolor*, biosynthesizing triketide α-pyrones for shuffling sulfate group during biosynthesis of caprazamycin antibiotics^32^. However, the ability of CPZ6 to form additional products was not investigated in this study. In an attempt to delineate the structural basis for MMAR_2470 enzymatic activity, we carefully compared the active sites of MMAR_2470 and MMAR_2474 proteins. While most of the cavity-lining residues were conserved in the proteins, variations were observed at certain positions. Using MMAR_2474 homology model as backbone, we systematically replaced side chains at *Phe*52, *Tyr*172, *Gly*189, *Trp*230, *Gln*238, *Leu*245 and *Val*283 of MMAR_2474 with *Leu*, *Phe*, *Ala*, *Tyr*, *Glu*, *Ile* and *Ile*, respectively, as present at equivalent positions in the MMAR_2470 protein. As can be observed from Fig. 5e, while these side chain changes drastically reduce cavity volume, docked methylated triketide α-pyrone fits well in the cavity. It is interesting to note that change from *Trp* to *Tyr* at position 230 exhibited hindrance and clashed with the three docked tetraketide reaction products (Fig. 5f). Presence of a *Tyr* at 230 position in MMAR_2470 protein likely impedes and limits elongation of the growing poly-β-keto chain to a triketide intermediate which is then cyclized and released as an alkyl-triketide α-pyrone product. Our product docking studies identify several residue positions that could play decisive roles in determining the catalytic products of the Mmar type III PKS proteins.

### Site-directed mutagenesis to investigate active site in MMAR_2474 protein

*In silico* product docking studies with the Mmar type III PKSs implicated several residue positions in playing important roles in determining and influencing the product profile. Product docking studies with MMAR_2474 protein revealed interactions of all three methylated tetraketide products with a specific Phe52 residue. The methylated tetraketide α-pyrone product showed additional interactions with *Tyr59* residue (Fig. 5b).

**Figure 6:**
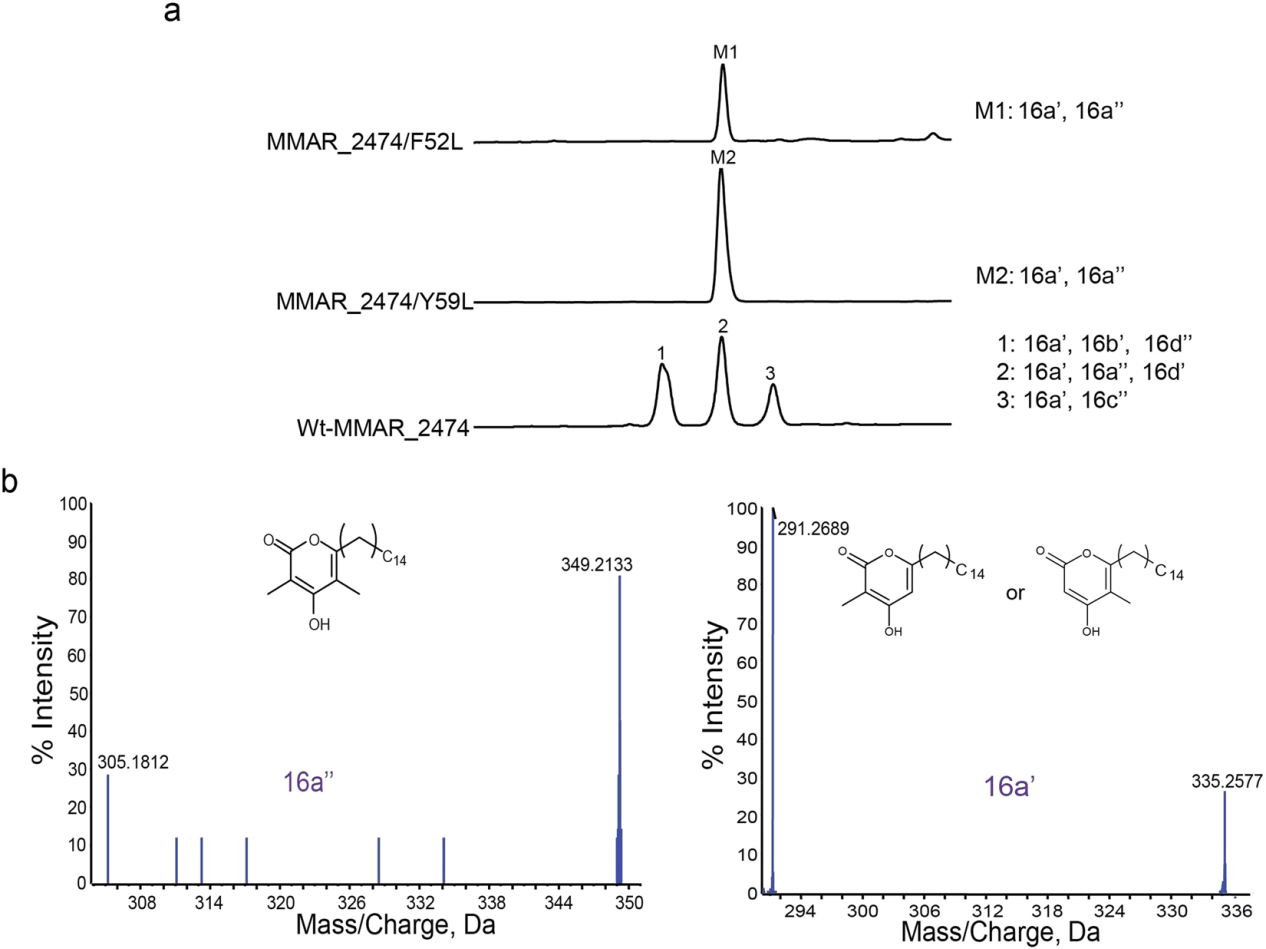
Biochemical characterization of F52L and Y59L mutants of MMAR_2474 protein. (**a**) UFLC chromatograms at 280 nm for reaction products of enzymatic assays carried out with palmitoyl-CoA (C_16_-CoA) as starter substrate and malonyl- and methylmalonyl-CoA as extenders. Reactions carried out with wild-type MMAR_2474 protein were used as control. (**b**) Tandem MS/MS analysis of reaction products of F52L and Y59L mutant proteins.

We attempted to investigate the role of these residues in stabilizing the tetraketide intermediate to form various methylated tetraketide products by site-directed mutagenesis. Both *Phe52* and *Tyr59* were mutated to *Leu* side chains as present in other homologous type III PKSs. Mutant proteins were expressed in *E. coli* using wild-type MMAR_2474 (wt-MMAR_2474) protein expression conditions. Purified F52L and Y59L mutant proteins were independently assayed for product formation using palmitoyl-CoA (C_16_-CoA) as starter substrate and a combination of malonyl-CoA and methylmalonyl-CoA as extender substrates. Fig. 6a illustrates the UFLC chromatogram of separated reaction products. As can be visualized, reactions with the mutant proteins displayed an altered product profile resulting in single product peaks (M1 or M2) in both the chromatograms. Peaks M1 and M2 corresponded to peak 2 of the chromatogram for wt-MMAR_2474 protein suggesting possible presence of the products 16a’, 16a” and 16d’ as identified above in Fig. 3. HRMS analyses of both M1 and M2 peaks, revealed molecular ions of [M-H]^−^ at *m/z* 335.2577 and *m/z* 349.2133 corresponding to mono- and di-methylated palmitoyl-triketide α-pyrones, 16a’ and 16a”, respectively. Identity of the reaction products was further confirmed by tandem MS/MS analyses (Fig. 6b). Notably, both the mutant proteins were impaired in catalyzing biosynthesis of tetraketide products. Biochemical characterization of mutant proteins corroborated our *in silico* findings and established a catalytically important role of *Phe52* and *Tyr59* side chains for tetraketide product formation in MMAR_2474 protein.

### Probing physiological importance of MMAR_2470 and MMAR_2474 proteins

**Figure 7:**
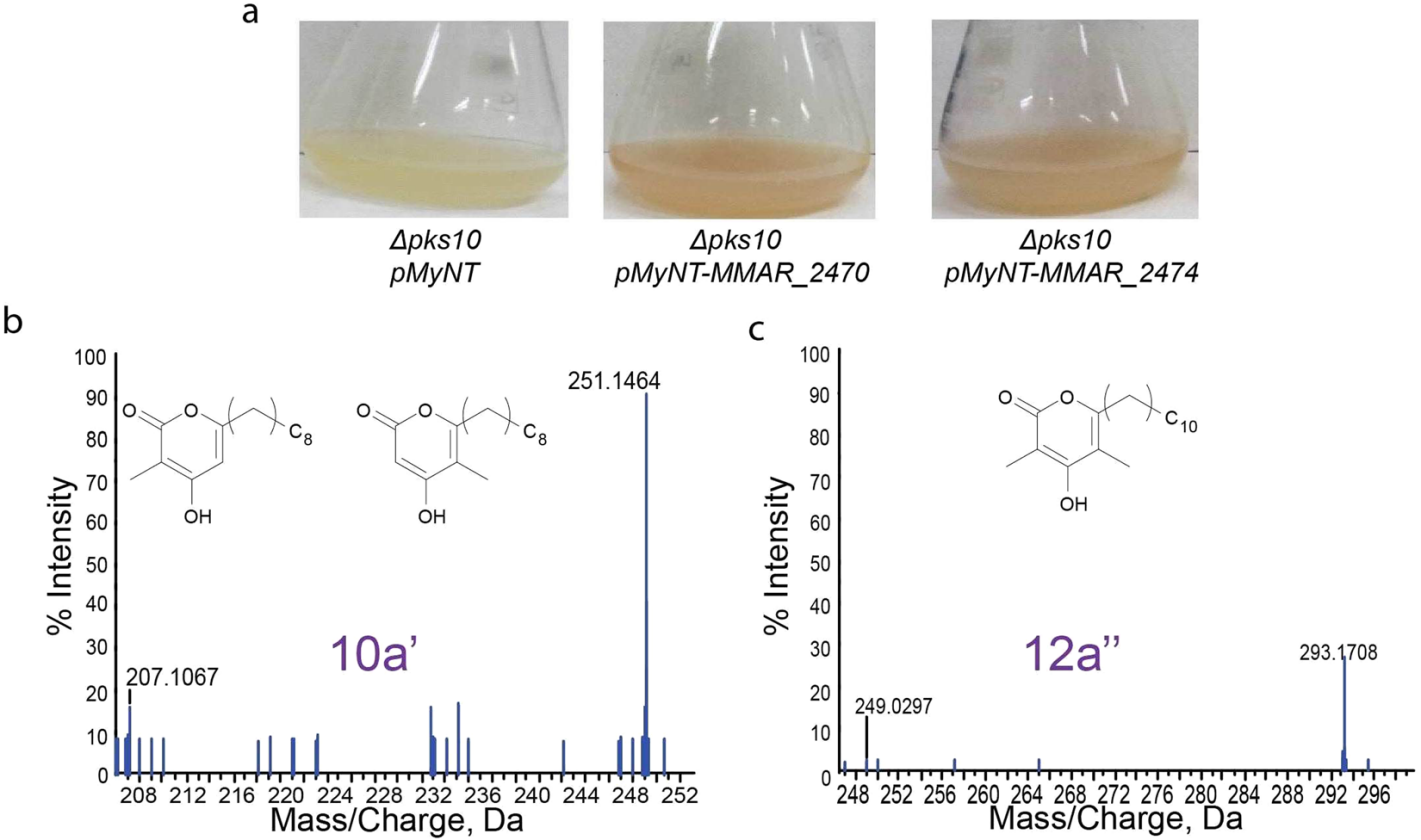
Over-expression of MMAR_2470 and MMAR_2474 proteins in *∆pks10 Mycobacterium smegmatis* strain. (**a**) Planktonic cultures over-expressing MMAR_2470 and MMAR_2474 proteins show brownish coloration. Triple-TOF HRMS metabolomic analysis of extracted culture filtrates of planktonic cultures over-expressing the Mmar type III PKSs identified (**b**) mono- and (**c**) di-methylated alkyl-triketide α-pyrones.

Mycobacterial MMAR_2470 and MMAR_2474 proteins belong exclusively to the pathogenic mycobacterial species. Interestingly, while MMAR_2470 shows a restricted product profile, the MMAR_2474 protein could catalyze the formation of methylated alkyl-resorcinol and methylated acyl-phloroglucinol products from the same catalytic pocket. We investigated the physiological relevance of the two mycobacterial type III PKS proteins by independently over-expressing these in a genetically modified, type III *pks* knockout strain (Δ*pks10*) of Msmeg. Msmeg genome harbors a single type III *pks* gene, *pks10*, in a three-gene operon. In a recent study, PKS10 protein from Msmeg was characterized to biosynthesize long-chain alkyl-resorcinols, which upon *o*-methylation and oxidation, led to the formation of novel polyketide quinones. These molecules facilitate mycobacterial survival in oxygen-deficient niches by participating in electron transport during anaerobic respiration. *mmar_2470* and *mmar_2474* genes were cloned in inducible mycobacterial expression vector *pMyNT* and expressed in Δ*pks10* Msmeg mutant strain^36^. Planktonic cultures over-expressing Mmar type III PKSs displayed brownish coloration upon induction with acetamide (Fig. 7a). Filtrates of these cultures were extracted and subjected to HRMS metabolomic analyses. As can be seen in Fig. 7b,c, tandem MS/MS analyses identified mono- and di-methylated alkyl-triketide α-pyrone products, respectively, in extracts of *Δpks10* Msmeg cultures over-expressing the MMAR_2470 and MMAR_2474 proteins (Supplementary Tables S2, S3).

**Figure 8:**
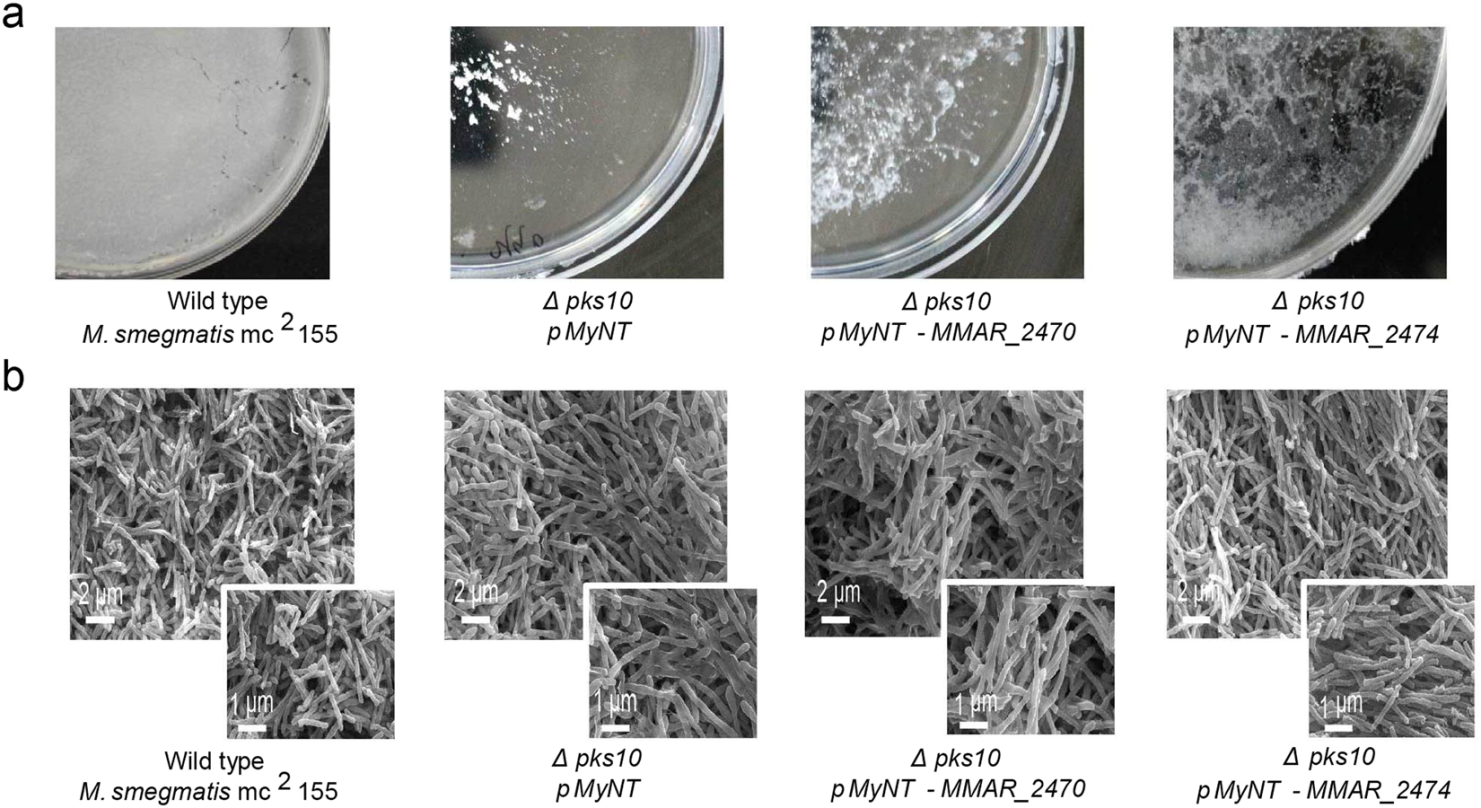
Biofilm development of ∆*pks10 Mycobacterium smegmatis* strains complemented with Mmar type III PKSs. (**a**) Pellicle development and, (**b**) scanning electron microscopy (SEM) of biofilms of wild-type *M. smegmatis* (Msmeg), ∆*pks10*-*pMyNT* Msmeg, ∆*pks10*-*pMyNT*-MMAR_2470 Msmeg and ∆*pks10*-*pMyNT*-MMAR_2474 Msmeg strains.

We further probed a possible role of the Mmar type III PKS proteins in facilitating mycobacterial growth in biofilms. Mycobacteria are naturally known to exist as stationary aggregates in biofilms. Development of biofilm pellicle was carried out for *Δpks10* Msmeg strains over-expressing *pMyNT*-MMAR_2470 or *pMyNT*-MMAR_2474 proteins and analyzed by scanning electron microscopy (SEM). As reported earlier, the mutant *Δpks10* Msmeg strain produced a fragile pellicle as compared to the reticulated architecture of the wild-type Msmeg biofilm^36^. As a compelling observation, over-expression of MMAR_2474 protein in *Δpks10* Msmeg mutant strain restored the porous community structure of the biofilm to moderately resemble the wild-type Msmeg pellicle (Fig. 8). Similar restoration of the biofilm phenotype has been reported by Anand *et al*., upon complementation of the *Δpks10* Msmeg biofilms with alkyl-benzoquinones generated from modification of alkyl-resorcinol product of PKS10 protein in Msmeg^36^. As MMAR_2474 protein biosynthesizes methylated alkyl-resorcinol products, we investigated the presence of polyketide quinones in the biofilm of *Δpks10*-*pMyNT*-MMAR_2474 strain by analyzing the biofilm pellicle extracts by high resolution metabolomics analyses. However, despite best efforts, we could not observe the quinone molecules. Over-expression of MMAR_2470 protein in the *Δpks10* Msmeg strain could not regenerate the biofilm pellicle, owing probably to the absence of methylated alkyl-resorcinol products in this strain. Our results suggest physiological importance of type III polyketide metabolites and implicate Mmar type III PKSs in assisting mycobacterial survival in stationary aggregates.

## Discussion

Mycobacterial polyketide gene clusters are involved in the biosynthesis of architecturally and functionally unique lipids that play major roles as effectors of virulence^6,48^. Several studies have provided understanding on PKS mediated mechanisms employed by pathogenic mycobacterial species to armor against the host defenses and establish virulent pathology. Efforts, however, are on to identify factors that determine persistence and dormancy in mycobacteria^8–10^. The existence of mycobacteria in self-assembled multi-cellular and structurally robust biofilms in host provides unique advantages of antimicrobial resistance^49^, host immune modulation^50^, and persistence^8,51^. Type III polyketide synthase genomic clusters from several bacteria have been lately associated with diverse bio-functionalities including, conferring antibiotic resistance in *Streptomyces*^18^, development of exine in dormant *Azotobacter* cells^35^, and survival capability of mycobacteria in anaerobic biofilms^36^.

In this study, we have functionally characterized two novel type III PKSs, MMAR_2470 and MMAR_2474 proteins from *Mycobacterium marinum* (Mmar) by a combination of computational, biochemical, mutational and physiological studies. Genes for both these proteins are present in a unique 2x type III *pks* genomic cluster, that includes genes for other type I modular/iterative PKSs. Presence of two type III *pks*s in a single genomic cluster has been previously reported only in the case of *ars* cluster in *Azotobacter*^35^. Our comparative genome analyses revealed a conservation of Mmar 2x type III *pks* cluster exclusively in the genomes of pathogenic mycobacterial species (Supplementary Table S5). It is interesting to note that this genomic organization is not observed in any other genera outside *Mycobacterium*, suggesting an extremely specialized function of the cluster in mycobacterial pathogenesis. Functional characterization of MMAR_2470 and MMAR_2474 proteins corroborated our phylogenetic findings and both the proteins could accept malonyl-CoA as well as methylmalonyl-CoA to biosynthesize mono- and di-methylated polyketide products (Supplementary Figure S3). MMAR_2474 exceptionally displayed the capacity to catalyze concomitant biosynthesis of methylated acyl-phloroglucinols and methylated alkyl-resorcinols. Our three-dimensional homology modeling and product docking studies identified interactions of tetraketide products with specific *Phe*52 and *Tyr*59 residues uniquely positioned in the substrate-binding tunnel in MMAR_2474 protein. Systematic site-directed mutagenesis studies revealed the inability of F52L and Y59L mutant proteins to synthesize tetraketide products, establishing their involvement in dictating the product profile of MMAR_2474 protein.

Mycobacterial pathogenicity is associated with the ability of the pathogen to grow and differentiate in multi-cellular assemblages, enveloped in extracellular matrix that provides a secure antimicrobial-resistant niche for propagation and development of persistence^37,49,52,53^. Alkyl-resorcinols from PKS10 in Msmeg, upon *o*-methylation and oxidation, form polyketide quinones that have been recently shown to facilitate growth and survival in anaerobic biofilms. Notably, over-expression of MMAR_2474 protein in a biofilm-growth defective *Δpks10* Msmeg mutant strain revived growth in stationary biofilms. While the polyketide quinones could not be detected in the extracts of revived biofilms, the biosynthetic activity of the over-expressing MMAR_2474 protein likely provided the alkyl-resorcinol precursor needed for quinone formation in the revived biofilms. Orthologous PKS11 protein in Mtb biosynthesizes similar precursors and has been implicated in biofilm generation^36^. Investigations in this study unravel a unique 2x type III *pks* genomic organization exclusively conserved in pathogenic mycobacterial species and emphasize the importance of type III polyketide synthases in aiding mycobacterial survival in complex communities. Our findings reveal unique insights on biosynthetic potentials of type III polyketide synthases from *M. marinum* and identify a new class of type III PKSs conserved in pathogenic mycobacteria.

## Methods

### Bacterial strains and materials

*Escherichia coli* XL-1 blue and BL21 (DE3) strains were used as cloning and expression strains, respectively. The sequenced *Mycobacterium marinum* (strain ATCC BAA-535/M) was kindly provided by Y. Singh (IGIB, India) and was used for genomic DNA isolation. The type III *pks* knockout, a *Δpks10* strain of *Mycobacterium smegmatis* mc^2^155 was a kind gift from R. S. Gokhale (NII, India) and was used for over-expressing *M. marinum* type III PKSs. The mycobacterial expression vector, *pMyNT* was kindly donated by Annabel Parret and Matthias Wilmanns (Addgene plasmid # 42191). Acyl-CoA starters and extender substrates were procured from Sigma. Middlebrook 7H9, Middlebrook 7H11 media and Sauton’s Fluid Media Base were purchased from Himedia. Restriction endonucleases and PCR master mix were purchased from New England Biolabs while PCR cleanup kit and Ni^2+^-NTA was purchased from Qiagen. Quik Change site-directed mutagenesis-XL kit was procured from Stratagene. All analytical, HPLC and MS grade solvents were purchased from Merck and Sigma.

### Cloning, expression, and purification of MMAR_2470 and MMAR_2474 proteins

Type III PKS genes, *mmar_2470* and *mmar_2474*, were amplified by PCR from genomic DNA of *M. marinum* using gene-specific forward primers (5′TTCATATGAGCGTCATCGCAGGTGTG3′) and (5′TTCATATGAGCGTTATCGCAGGTGTG3′) for *mmar_2470* and *mmar_2474*, respectively, containing NdeI restriction enzyme site and reverse primers (5′GGGAATTCCCGTGCCAACGCAGCAGTACTAG3′) and (5′AAGAATTCCCGTGCCAGCGCAGTAACACCAG3′) for *mmar_2470* and *mmar_2474*, respectively, containing EcoRI restriction enzyme site and cloned into pBluescript SK (+) (Stratagene). The authenticity of clones was confirmed by automated nucleotide sequencing. These *mmar_2470* and *mmar_2474*genes were sub-cloned into a *pET28c* (Novagen) and *pET21c* (Novagen) expression vectors, respectively for protein purification. The MMAR_2470 protein was expressed as both N- and C- terminal hexa-histidine-tagged protein whereas MMAR_2474 was expressed as C- terminal hexa-histidine-tagged protein in the BL21/ (DE-3) strain of *E. coli*. The recombinant *E. coli* cells harboring the expression plasmids were grown in Luria Bertini broth at 30 °C until an OD 600 of 0.5 units was reached. The culture was induced with 0.5 mM isopropyl-1-thio-β-D galactopyranoside (IPTG) and further incubated in a shaker at 22 °C for 16–20 h. After harvesting, the cells were re-suspended in lysis buffer (50 mM Tris pH 8.0, 10% glycerol, 0.15 M NaCl) and disrupted using 8 cycles of sonication at 30% amplitude. Cell debris was removed by centrifugation at 13,000 rpm for 30 min at 4 °C. 1 ml of Ni^2+^-NTA slurry per liter of culture was added to the supernatant and was incubated at 4 °C for 1 hour. The mixture was loaded onto a column using gravity flow. The resin was washed with wash buffer (50 mM Tris pH 8.0, 10% glycerol) till the unbound proteins were removed. The protein was eluted using elution buffers containing an increasing concentration of imidazole (5 mM to 250 mM). Expression and purification of both MMAR_2470 and MMAR_2474 proteins were performed using modified protocol mentioned in earlier literature^27,36^.

### Enzymatic assay and product characterization

The standard reaction was carried out using 100 μM starter CoA and 50 μM malonyl-CoA and/or 50 μM methylmalonyl-CoA extender with 50μg of protein at 30 °C for 120 min. The reaction was quenched with 5% acetic acid. Products were extracted with 2x 300 μl ethyl acetate and dried under vacuum. Products from at least three reactions were pooled after vacuum drying by dissolving in 20 μl of methanol. Products were resolved on an analytical C5-reverse phase-UFLC column using a 5–100% gradient of acetonitrile containing 1% acetic acid for 60 min. Resolved products were further characterized using SCIEX Triple-Time Of Flight (Triple-TOF) 6600 HRMS.

### Site-Directed Mutagenesis

Site-directed mutagenesis was carried out using Quik Change site-directed mutagenesis-XL kit from Stratagene. The recombinant plasmid containing the wild-type *mmar_2474* gene was used as the DNA template. The plasmids containing the mutant genes were used to transform *E. coli* BL21 (DE3), and recombinant colonies were used for protein expression. Mutant proteins were expressed and purified using experimental conditions same as used for wild-type MMAR_2474 protein.

### *M. marinum* type III *pks* complementation of *∆pks10 Mycobacterium smegmatis* mutant strain and metabolomic analyses of planktonic culture and biofilm extracts

The *mmar_2470* and *mmar_2474* genes were cloned into a mycobacterial expression vector, *pMyNT*, using NdeI site. The *∆pks10* mutant strain of *M. smegmatis* mc^2^155^36^ was electroporated with *pMyNT*-*mmar_2470* and *pMyNT*-*mmar_2474* plasmids and used for over-expression of the *M. marinum* type III PKSs. Transformed colonies were screened in 7H11 media with hygromycin and kanamycin as selectable markers. Planktonic cultures were grown in 7H9 media supplemented with ADC, 0.18% glycerol and 0.05% tween 80 with 100 µg/ml hygromycin and 50 µg/ml kanamycin and were induced with 1 mM acetamide when the OD at 600 nm reached 0.8. Biofilm pellicles were grown in Sauton’s media supplemented with 2% glucose and 2% glycerol and with 100 µg/ml hygromycin and 50 µg/ml kanamycin and induced with 1 mM acetamide at the start of the culture and incubated at 37 °C for 14 days. Cells from grown planktonic culture and biofilm pellicle were harvested and resuspended in 100 mM Tris-Cl, pH 8.0. After resuspension the culture supernatants and pellicle were acidified with acetic acid to pH 4 and polyketide derived lipid molecules were extracted by adding a double volume of ethyl acetate. The organic layer containing the extracted products was separated and concentrated using multi-vapor. The extracts were dissolved in methanol and products were characterized using SCIEX Triple-TOF 6600 HRMS using multiple-reaction-monitoring (MRM) and information dependent acquisition (IDA) methods.

### Scanning electron microscopy

Scanning electron microscopy was performed for the biofilm pellicle samples using standard protocols. Briefly, the pellicle was fixed over-night with a fixative containing 2.5% (vol/vol) glutaraldehyde and 4% paraformaldehyde and washed with sodium cacodylate buffer at room temperature. The pellicle was then osmicated with 1% Osmium tetroxide (wt/vol) in 0.1 M sodium cacodylate buffer for 40 min at room temperature in dark and washed with milli-Q water at room temperature. Pellicle was then gradually dehydrated with increasing concentrations of ethanol followed for 15 min and incubated in dark with hexamethyldisilazane (HMDS). The samples were mounted on stubs, sputter coated with gold particles for 300 s, and imaged on a Zeiss-EVO LS15 Scanning Electron Microscope (Carl Zeiss).

### *In silico* studies

The sequences of mycobacterial type III PKS clusters were retrieved in FASTA format from UniProt. Subsequently, a homology search was done using NCBI BLASTp program^54^ to identify potentially related proteins. Cluster conservation was identified using UniProt and microbrowser search. Experimentally characterized type III PKS amino acid sequences were downloaded from UniProt. The phylogenetic tree was constructed using maximum likelihood method in MEGA7^55^.

Homology modeling of type III PKSs was carried out using Biovia Discovery Studio version 4.5. FtpA, MMAR_2470, MMAR_2474 and SrsA proteins were modeled using a 4JAO_D^42^ template whereas DpgA model was generated using the 1TED_B^21^ template (Supplementary Table S1). 1U0M_B^20^ was used for PhlD model preparation (Supplementary Table S1). Ligand/product binding pockets and respective cavity volumes of homology models of type III PKSs were identified using Biovia Discovery Studio 4.5.

Predicted-ligand/product library was generated using ChemDraw software and ligands/products were prepared in Biovia Discovery Studio 4.5 along with protein preparation for protein-ligand/product docking studies. Protein ligand/product docking was done using CDOCKER of Biovia Discovery Studio 4.5. CDOCKER is the CHARMm-based docking method that generates highly accurate docked poses^56^.

### Data availability

All data generated or analysed during this study are available from the corresponding author on reasonable request.

## Electronic supplementary material

Supplementary Information
