# Supplementary material for: Novel Type III Polyketide Synthases Biosynthesize Methylated Polyketides in Mycobacterium marinum
Source: Sci Rep. 2018 Apr 25;8:6529. doi: 10.1038/s41598-018-24980-1 (PMC5916927; doi:10.1038/s41598-018-24980-1)
Supplement: Supplementary file 1 — Supplementary Information [file 41598_2018_24980_MOESM1_ESM.docx]

**Supplementary Information**

**Novel Type III Polyketide Synthases Biosynthesize Methylated Polyketides in *Mycobacterium marinum***

**Amreesh Parvez^1^, Samir Giri^1,2^, Gorkha Raj Giri, Monika Kumari^3^, Renu Bisht and Priti Saxena***

Chemical Biology Group, Faculty of Life Sciences and Biotechnology, South Asian University, New Delhi, 110021, India

*Correspondence: [psaxena@sau.ac.in](mailto:psaxena@sau.ac.in)

**^1^**these authors contributed equally to this work

**^2^**Present address: Department of Ecology, School of Biology, University of Osnabrück, Osnabrück, 49076, Germany

**^3^**Present address: Department of Biochemistry, University College of Medical Sciences, Delhi, 110095, India

**Supplementary Figure S1**


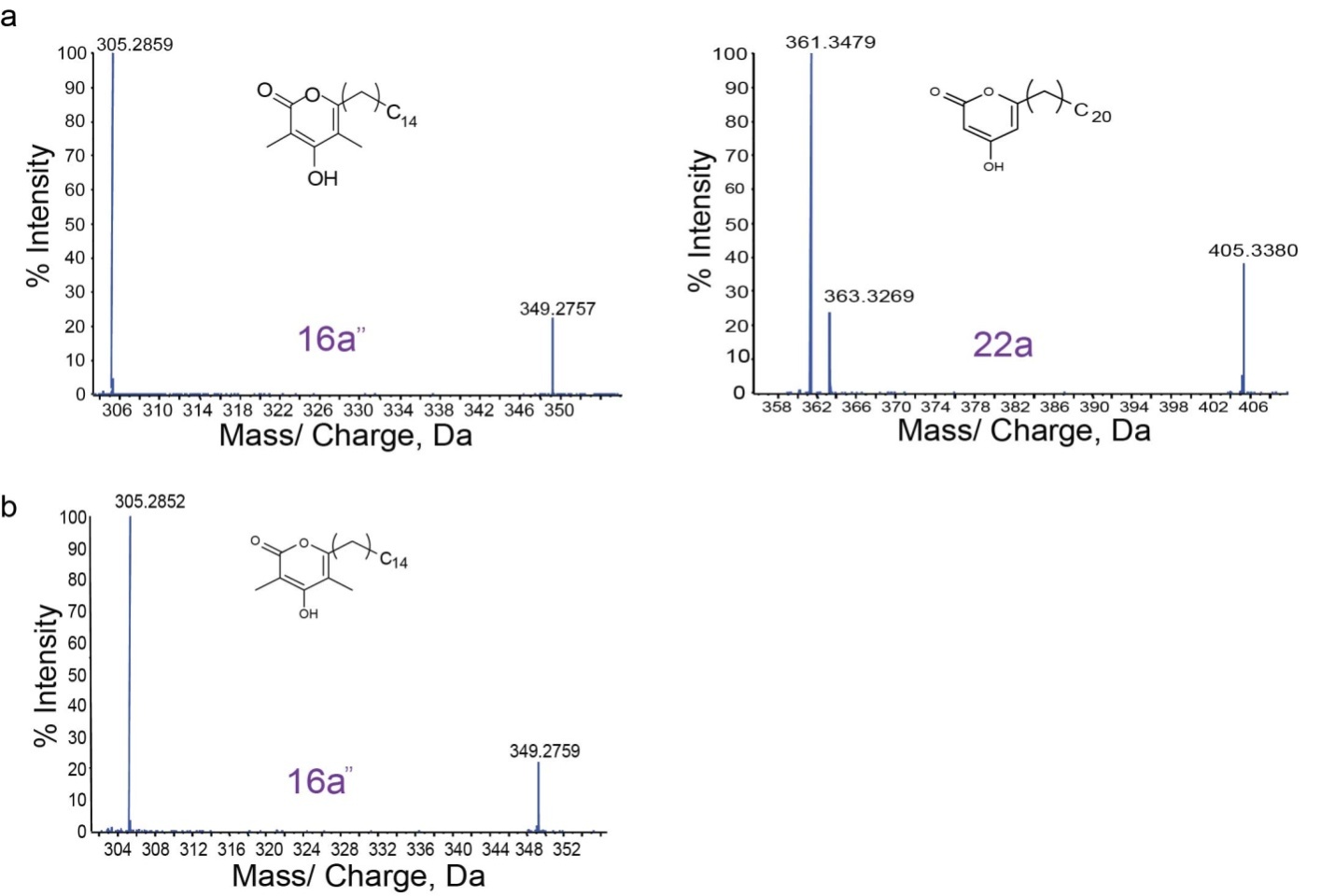


**Supplementary Figure S1: Tandem MS/MS profiles for *in vitro* reaction products of the mycobacterial type III PKSs.** (a) products of MMAR_2470 protein and (b) products of MMAR_2474 protein.

**Supplementary Figure S2**

**Supplementary Figure S2:Multiple Sequence Alignment of bacterial type III PKSs.** Catalytic triad is in *blue*, product binding site residues are in *red*, extender substrate binding site residues are in *green*, residues determining cavity volume are in *purple* and residues present in *brown* are common for the extender as well as product binding sites.

**Supplementary Figure S3**

**Supplementary Figure S3: Reactions catalyzed by mycobacterial type III PKSs.** Starter(s) refers to the priming acyl-CoAs. Malonyl-CoA is represented as MCoA and methylmalonyl-CoA is referred to as MMCoA. The scheme represents possible reactions and products that can be obtained with the two extender units. (a) Represents reactions and products with MCoA as an extender, (b) Represents reactions and products with 1x MCoAand 1x MMCoA as extenders, (c) Represents reactions and products with 2x MCoA and 1x MMCoA as extenders, (d) Represents reactions and products with 2x MMCoA as an extender (e) Represents reactions and products with 1x MCoA and 2x MMCoA as extenders. The non-methylated products, triketide α-pyrones are represented as **a**, tetraketide α-pyrones as **b**, alkyl-resorcinols as **c** and acyl-phloroglucinols as **d**. Mono-methylated variants of **a**, **b**, **c**, and **d** are represented as **a’**, **b’**, **c’** and **d’**, respectively. Di-methylated variants of **a**, **b**, **c**, and **d** are represented as **a’’**, **b’’**, **c’’** and **d’’**, respectively. Structures boxed with *black* broken line are chemically not feasible. Products boxed with *magenta* broken lines are biosynthesized by MMAR_2470 protein, in *blue* are biosynthesized by MMAR_2474 protein and in *orange* are biosynthesized by both the proteins.

**Supplementary Table S1: Details of templates used and energy scores in Homology modeling**

| **Protein** | **Template** | **% Identity** | **DOPE Score** |
| --- | --- | --- | --- |
| DpgA | 1TED_B | 26.82 | -37,089.14 |
| PhlD | 1U0M_B | 46.53 | -41317.1 |
| SrsA | 4JAO_B | 49.43 | -42,435.30 |
| FtpA | 4JAO_B | 48.13 | -42412.9 |
| MMAR_2474 | 4JAO_B | 84.38 | -44,650.00 |
| MMAR_2470 | 4JAO_B | 73.37 | -43,844 |

**Supplementary Table S2: IUPAC names and chemical structures of the compounds identified by MS/MS analyses in MMAR_2470 *in vitro* assays and metabolomics**

| **S. No.** | **Molecules and IUPAC name** | **Compounds identified by MS/MS** |
| --- | --- | --- |
| **1.** | 4-hydroxy-3-methyl-6-pentadecyl-pyran-2-one  4-hydroxy-5-methyl-6-pentadecyl-pyran-2-one  O  **OH**  **O**  C  14 | **(16a') Mono-methylated palmitoyl-triketide alpha-pyrone** MS/MS fragments: 335.2577, 291.2689 m/z  Identified in MMAR_2470 Biochemical Assays with MMCoA and MCoA extender |
| **2.** | 4-hydroxy-3,5 dimethyl-6-pentadecyl-pyran-2-one  O  **OH**  **O**  C  14 | **(16a'') Dimethylatedpalmitoyl-triketide alpha-**  **Pyrone**  MS/MS fragments: 349.2133, 305.1821 m/z Identified in MMAR_2470 Biochemical Assays with MMCoA and MCoA extender |
| **3.** | 4-hydroxy-3,5 dimethyl-6-pentadecyl-pyran-2-one  O  **OH**  **O**  C  14 | **(16a'') Dimethylatedpalmitoyl-triketide alpha-pyrone**  MS/MS fragments: 349.22757, 305.2859 m/z. Identified in MMAR_2470 Biochemical Assays with MmCoA extender |
| **4.** | 6-henicosyl- 4-hydroxy- pyran-2-one | **(22a) Docosanyl-triketide alpha Pyrone**  MS/MS fragments: 405.3380, 361.3479 m/z Identified in MMAR_2470 Biochemical Assays with MCoA extender |
| **5.** | 6-decyl-4-hydroxy- 3-methyl-pyran-2-one      6-decyl-4-hydroxy- 5-methyl-pyran-2-one | **(10a') Monomethylatedldecanoyltriketide**  **alpha-pyrone**  MS/MS fragments: 251.1464, 207.1067 m/z Identified in MMAR_2470 Metabolomics |

**Supplementary Table S3: IUPAC names and chemical structures of the compounds identified by MS/MS analyses in MMAR_2474 *in vitro* assays and metabolomics**

| **S. No.** | **Molecules and IUPAC Names** | **Details** |
| --- | --- | --- |
| **1.** | O  **OH**  **O**  C  14  4- hydroxy-3-methyl-6-pentadecyl-pyran-2-one  O  **OH**  **O**  C  14  4-hydroxy-5-methyl-6-pentadecyl-pyran-2-one | **(16a')Monomethylatedpalmitoyl-triketide alpha-pyrone**  MS/MS fragments: 335.2593, 291.2695 *m/z*  Identified in MMAR_2474 Biochemical Assays with MMCoA and MCoA extender |
| **2.** | O  **OH**  **O**  C  14  4-hydroxy-3,5-dimethyl-6-pentadecyl-pyran-2-one | **(16a'')Dimethylatedpalmitoyl-triketide alpha-pyrone**  MS/MS fragments: 349.2759, 305.2852 m/z  Identified in MMAR_2474 Biochemical Assays with MMCoA extender |
| **3.** | O  **O**  **O**  **OH**  C  14  4-hydroxy-5-methyl-6-(2 oxohexadecyl) pyran-2-one  O  O  **OH**  O  C  14  4-hydroxy-6-(1-methyl-2-oxo-hexadecyl) pyran-2-one  O  **OH**  **O**  **O**  C  13  4-hydroxy-3-methyl-6-(2-oxohexadecyl) pyran-2-one | **(16b')Monomethylatedpalmitoyl-tetraketide alpha- pyrone**  MS/MS fragments: 377.2706,333.2804, 139.0406 m/z  Identified in MMAR_2474 Biochemical Assays with MMCoA and MCoA extender |
| **4.** | **OH**  **HO**  **OH**  **O**  C  13  1-(2,4,6-trihydroxy-3-methyl-phenyl)hexadecan-1-one  **OH**  **HO**  **OH**  **O**  C  13  1-(2,4,6-trihydroxy-3-methyl-phenyl)hexadecan-1-one | **(16d')Monomethylatedpalmitoyl-phloroglucinol**  MS/MS fragments: 377.2694,333.2796, 139.0411 m/z  Identified in MMAR_2474 Biochemical Assays with MMCoA and MCoA extender |

**Supplementary Table S3 continued**

**Supplementary Table S3: IUPAC names and chemical structures of the compounds identified by MS/MS analyses in MMAR_2474 *in vitro* assay and metabolomics**

| **S. No.** | **Molecules and IUPAC Names** | **Details** |
| --- | --- | --- |
| **5.** | O  **OH**  **O**  C  14  4-hydroxy-3,5-dimethyl-6-pentadecyl-pyran-2-one | **(16a'')Dimethylatedpalmitoyl-tetraketide alpha-pyrone**  MS/MS fragments: 349.2443,305.2846 *m/z*  Identified in MMAR_2474 Biochemical Assays with MMCoA and MCoA extender |
| **6.** | **OH**  **HO**  C  14  2,4-dimethyl-5-pentadecyl-benzene-1,3-diol  **HO**  **OH**  C  14  4,6-dimethyl-5-pentadecyl-benzene-1,3-diol | **(16c'')Dimethylatedpalmitoyl-Resorcinol**  MS/MS fragments: 347.2589, 305.2471, 303.1949 *m/z*  Identified in MMAR_2474 Biochemical Assays with MMCoA and MCoA extender |
| **7.** | **OH**  **HO**  **OH**  **O**  C  13  1-(2,4,6-trihydroxy-3,5-dimethyl-phenyl)hexadecan-1-one | **(16d'')Dimethylatedpalmitoyl-Phloroglucinol**  MS/MS fragments: 391.2861, 347.2963, 153.0559 *m/z*  Identified in MMAR_2474 Biochemical Assays with MMCoA and MCoA extender |
| **8.** | O  **OH**  **O**  C  10  6-dodecyl-4-hydroxy-3,5-dimethyl-pyran-2-one | **(12b'')Monomethylatedlauroyltriketide alpha-pyrone**  MS/MS fragments: 293.1708, 249.0297 m/z  Identified in MMAR_2474 metabolomics |

**Supplementary Table S4: MMAR_2474 docking with methylated polyketide products**

**Supplementary Table S5: Pathogenic mycobacterial species harboring 2x Type III *pks*s cluster**

| **S.No.** | **Mycobacterial species** |
| --- | --- |
|  | *Mycobacterium pseudoshottsii* |
|  | *Mycobacterium gastri* |
|  | *Mycobacterium bovis* |
|  | *Mycobacterium intracellulare* |
|  | *Mycobacterium tuberculosis* |
|  | *Mycobacterium haemophilum* |
|  | *Mycobacterium marinum* |
|  | *Mycobacterium yongonense* |
|  | *Mycobacterium caprae* |
|  | *Mycobacterium chimaera* |
|  | *Mycobacterium microti* |
|  | *Mycobacterium colombiense* |
|  | *Mycobacterium africanum* |
|  | *Mycobacterium paratuberculosis* |
|  | *Mycobacterium kansasii* |
|  | *Mycobacterium lentiflavum* |
|  | *Mycobacterium canettii* |
|  | *Mycobacterium orygis* |
|  | *Mycobacterium gordonae* |
|  | *Mycobacterium ulcerans* |
|  | *Mycobacterium europaeum* |
|  | *Mycobacterium indicuspranii* |
|  | *Mycobacterium liflandii* |
|  | *Mycobacterium bohemicum* |
|  | *Mycobacterium triplex* |
|  | *Mycobacterium parascrofulaceum* |
|  | *Mycobacterium avium* |
